# Saying YES to Careers in Cancer Research: A Collaborative Model for Evaluating and Improving a Cancer Education and Research Program

**DOI:** 10.18103/mra.v13i4.6432

**Published:** 2025-04-30

**Authors:** James A. Render, Ever Mkonyi, Ashley Langford, Kelli Qua, Damian J. Junk

**Affiliations:** 1 Case Comprehensive Cancer Center, School of Medicine, Case Western Reserve University, Cleveland, OH, USA; 2 Center for Medical Education, School of Medicine, Case Western Reserve University, Cleveland, OH, USA

## Abstract

The Case Comprehensive Cancer Center’s Youth Enjoy Science (YES) Program is funded by the National Cancer Institute to promote broad interest in pursuing a career in biomedical research via early intervention strategies that inspire student interest, help envision research as a career path, and strengthen practical research and career skills. A major aspect of the YES program is an 8-week immersion into cancer center faculty laboratories to conduct a cancer research project over the summer. The overall goal is to excite students to consider a future career in biomedical research or healthcare to enhance the future cancer-focused workforce. To ensure the YES program is meeting its goals and providing an exceptional experience for students, an external evaluation team performs an annual evaluation that incorporates a mixed methods approach combining mechanisms for formal and informal feedback. The team shares key insights with program leadership, faculty, and student representatives to ensure that all feedback directly informs program adjustments. This approach emphasizes actionable results rather than passive data collection, reinforcing the program’s commitment to continuous quality improvement. Formal surveys suggest the program has been consistently meeting its goals of increasing student understanding of cancer biology and research. However, a recent reduction in survey response rates makes it challenging to determine whether survey respondents are primarily those with the most positive experiences. Integrating both formal and informal feedback mechanisms in program evaluation is essential for capturing the full range of student experiences. Thus, it is critical that training programs provide a channel for informal discussions. The YES program coordinator maintains close relationships with all stakeholders and is deeply familiar with student participants, from the application process to ongoing program activities. As an approachable and engaged leader, the coordinator effectively gathers feedback and implements changes to enhance program performance. In response to formal and informal feedback, the YES program curriculum has been modified to enhance not only student and mentor experiences but also the process for soliciting feedback in the future.

## Introduction

Efforts to diversify and expand the biomedical research workforce have increasingly focused on early intervention strategies that engage students in immersive research experiences.^1–5^ High school students, in particular, benefit from structured pathway programs that expose them to potential career options early in their academic journeys.^2,4,6^ Research suggests that providing high school students with hands-on exposure to scientific fields enhances their ability to make informed career choices, increases their self-efficacy in science, technology, engineering, and mathematics (STEM) disciplines, and fosters long-term engagement in research careers.^7–10^ Programs such as the Youth Enjoy Science (YES) initiative, funded by the National Cancer Institute, aim to inspire students to envision biomedical research as a viable career path while equipping them with foundational research and professional skills. A key component of the YES program is an intensive 8-week summer research experience in cancer center faculty laboratories, designed to foster interest in cancer-related research and healthcare careers. These types of initiatives have been shown to enhance students’ research competencies and develop career goals.^1,11–14^

Engaging high school students in pathway programs is particularly crucial in addressing differences in STEM participation, as disadvantaged students often lack access to research experiences and professional mentorship in their formative years.^1,12,13,15^ Early exposure to research not only broadens students’ understanding of potential careers but also helps them develop critical thinking, problem-solving, and communication skills essential for success in higher education and beyond.^15^ Longitudinal studies have demonstrated that students participating in pathway programs are more likely to pursue STEM degrees and careers, reinforcing the importance of sustained mentorship and experiential learning opportunities.^2,6,8–10,15,16^

Program evaluation plays a critical role in ensuring that such initiatives achieve their intended outcomes. Mixed methods approaches that combine quantitative and qualitative feedback mechanisms have been widely recognized as effective in capturing the complexities of student experiences.^17,18^ Despite growing recognition of the need for robust evaluation strategies, gaps remain in understanding how best to incorporate feedback to enhance program effectiveness. This study examines how the integration of formal and informal evaluation approaches can strengthen program assessment, ensuring a more comprehensive understanding of student experiences and fostering ongoing program improvement.

### DESCRIPTION OF THE PROGRAMS

The YES program is a partnership between the Case Comprehensive Cancer Center (Case CCC), the economically challenged Cleveland Metropolitan and East Cleveland School Districts, and greater Cleveland area urban and suburban schools. The Case CCC is a partnership among Case Western Reserve University (CWRU), Cleveland Clinic, and University Hospitals that integrates basic, translational, clinical, and community-based cancer research throughout northeast Ohio, a region of urban manufacturing and rural agriculture. Importantly, the Case CCC promotes the education and training of the next generation of cancer-focused scientists and healthcare professionals. The purpose of YES, funded by the National Cancer Institute, is to promote broad interest in pursuing a career in biomedical research via early intervention strategies that inspire student interest, help envision research as a career path, and strengthen practical research and career skills. YES excites, engages, and immerses middle and high school students and teachers in STEM education and cancer research through 3 major activities: 1) Learn To Beat Cancer, 2) Teach To Beat Cancer, and 3) Research To Beat Cancer.^1,12–14^

### LEARN TO BEAT CANCER

The goal of Learn to Beat Cancer is to implement an intervention at an early age to engage, excite, educate, and encourage students from grades 6, 7, and 8, and their family members to learn principles of cancer biology, research, clinical treatment, and prevention. A series of 13 age-appropriate, active-learning experiences guided by medical student volunteers are provided to systematically and sequentially introduce cancer research and care to young students to initiate their pursuit of relevant education and career opportunities.

### TEACH TO BEAT CANCER

The goal of Teach to Beat Cancer is to: 1) immerse grade 6–12 science teachers in cancer research and education to enhance their skills, knowledge, and abilities to teach science and principles of cancer biology; 2) to develop a cancer-focused curriculum to take back to their classrooms that encourages cancer risk reduction activities; and 3) to partner with the Case CCC to motivate middle and high school students to pursue careers in cancer research and healthcare.^11^ YES teachers participate alongside high school students in an 8-week laboratory immersion explained below. In addition, the teachers meet weekly as a group for a unique teacher focused workshop that explores curriculum development and dissemination strategies, DNA damage and repair, genetic risks of cancer and pedigree analysis, hereditary cancer syndromes, immunotherapy and cancer vaccines, tobacco control, and other strategies for cancer prevention.

### RESEARCH TO BEAT CANCER

The central activity of Research to Beat Cancer is an 8-week *immersion in a cancer research project* selected to align with student interests mentored by faculty members of the Case CCC. Students are placed in a laboratory where they are introduced to the scientific method, laboratory techniques, the responsible conduct of research, and are encouraged to gradually develop an independent research question and project within the overall scope of the selected laboratory. The immersive research experience introduces the principles of scientific investigation, research techniques, focused knowledge, and relevance to the clinic to enhance the students’ interest and knowledge in cancer biology, research, and healthcare professions. Additional activities occur throughout each week of the program to provide additional scientific exploration, career development, and academic success.

*Lunch and Learn* and *Career Café* are discussions led by CWRU faculty and staff from a variety of research backgrounds that introduce students to emerging state-of-the-art scientific research and techniques as well as the multitude of career options within biomedical research and healthcare. These sessions also provide instruction to prepare for college testing, application, choice, and financing by inviting the CWRU undergraduate admissions staff, and the director of the CWRU MD/PhD program to introduce their programs and provide guidance for applicants.

*Near Peer Mentoring* provides a comfortable venue to encourage students to express and discuss their research within small groups. The small groups are led by CWRU graduate and medical students who are a few steps further in their academic training that act as inspirational role models and advocates. Discussions with near peer mentors specifically explore student stress, academic achievement beyond high school including graduate level scientific research, and maintaining student wellness and resiliency. Near peers also actively listen for any challenges the students may be facing in their laboratory immersion.

*Writing Workshops* are provided by the CWRU Writing Resource Center. The workshops are small group instructional classes provided to select students who require additional training in reading comprehension, writing, and presentation skills. The workshops focus on reading comprehension tools for searching scientific literature and preparation of scientific abstracts, research papers, and college application essays. An important aspect of the workshops is instructor-guided opportunities for students to read and critique each other’s work.

The goal of Research to Beat Cancer is to engage, excite, and encourage high school students to finish high school and college and pursue careers in cancer research and healthcare. It is a matrix of complementary activities that engage students in cancer-focused biomedical research, introduce a variety of careers in biomedical research and healthcare, and provide an educational foundation for future academic and career success. Evaluation of the activities that comprise Research to Beat Cancer is the focus of this manuscript.

## Methods

### PROGRAM EVALUATION

Evaluation of the YES Program follows a participatory and utilization-focused approach, ensuring that students, faculty, and program staff play an active role in shaping program improvements.^18–20^ Evaluation activities are conducted as a partnership between the Office of Medical Education Research and Evaluation (MERE), which is external to the Case CCC, and the YES program coordinator, faculty, and other key stakeholders. By incorporating a mixed methods approach that integrates formal and informal feedback mechanisms, the evaluation process prioritizes not only data collection but also triangulation and expansion which promote the direct application of findings to program development.^17,21^ Formal data collection includes structured pre- and post-program surveys and focus groups, while informal feedback is gathered through direct conversations and observational insights from program stakeholders. These complementary methods allow for responsiveness to student concerns and inform iterative curriculum modifications.

### PRE- AND POST-PROGRAM SURVEYS

Structured pre- and post-program surveys serve as a primary formal evaluation tool. Students complete surveys assessing their experiences, the effectiveness of program components, areas for improvement, and their future academic or career plans. Faculty preceptors complete a separate survey evaluating student preparedness, engagement, and growth throughout the program. All instruments incorporate Likert-scale, multiple-choice, rank-order, and free-text response formats to capture a comprehensive range of feedback. Given the evolving nature of the program, survey items are reviewed annually with input from various stakeholders to ensure relevance. MERE conducts the formal activities of YES program evaluation. Surveys are voluntary.

### FOCUS GROUPS

Semi-structured focus groups are conducted by MERE at the end of the program to provide deeper insights into student experiences. A standardized discussion guide is used, consisting of three core questions with additional probing questions to encourage elaboration. Topics include student engagement and feedback on program components, mentorship experiences, and future plans. Focus groups, facilitated by trained evaluators, occur during the final week of the program, allowing participants to reflect on their experiences.

### PROCEDURES

This study was reviewed and approved by the CWRU IRB (IRB #201920701). Surveys are an integrated component of the program evaluation process and are strongly encouraged as part of course participation; however, students (or their parents) can opt out of having their responses included in research analyses. Both survey and focus group participation are voluntary. Students, parents, and faculty receive copies of informed consent documents detailing the use of evaluation data for research purposes. Surveys are distributed via Qualtrics followed up by email reminders for non-respondents. Focus group volunteers are solicited via email and are conducted in person and recorded using a web-based transcript service. To ensure confidentiality, any participant information is removed from the dataset before analysis. After data collection is complete, all data are de-identified and reported in the aggregate.

### DATA ANALYSIS AND UTILIZATION

A mixed methods approach is used for data analysis. Quantitative survey data are analyzed using descriptive and inferential statistics in IBM SPSS Version 27, with Chi-square tests conducted to examine response distribution differences across program years, if applicable. Qualitative responses, including open-ended survey comments and focus group transcripts, are analyzed using an inductive and deductive content analysis approach in Atlas TI. A coding framework was developed based on survey items, focus group prompts, and the Standards for Reporting Qualitative Research.^22^ To enhance reliability, coding was reviewed by a second researcher unaffiliated with the YES Program. Findings are actively used to drive curriculum decisions, reflecting a utilization-focused evaluation model. The evaluation team shares key insights with program leadership, faculty, and student representatives to ensure that feedback directly informs program adjustments. This approach emphasizes actionable results rather than passive data collection, reinforcing the program’s commitment to continuous improvement.

### WRITING WORKSHOP PROGRAM EVALUATION

Similar to the MERE evaluation, students who opt in and are participants in the writing workshops are given pre- and post-program surveys by the director of the CWRU writing center. The YES Program Writing Workshop Assessment Report incorporates the results of the surveys as well as the curriculum overview, summary, and writing faculty feedback. The assessment report findings and recommendations are independent of MERE and based on each summer’s workshop experiences over a 3-year period and does not necessarily reflect the findings of the MERE survey. Student success is measured by the reporting of five student learning outcomes based on Likert-scale responses.

### INFORMAL FEEDBACK FROM MENTORS AND STUDENTS

The YES program coordinator is located on campus near the laboratories where students are primarily conducting research and meets the students for lunch during curricular activities 3 days a week. The physical proximity to labs combined with open lines of communication between the students, mentors, and coordinator enables feedback solicited from or provided by students and mentors allowing the coordinator to act on feedback when appropriate. Regular discussions with mentors and administrative staff further ensure that feedback is actively integrated into decision-making. Thus, in-person conversation plays a critical role in engaging with stakeholders, documenting concerns, and advocating for curriculum modifications based on feedback. This participatory evaluation process strengthens stakeholder investment in program improvements and allows for adaptations to address emerging needs. This hands-on approach maximizes a positive experience for the student(s) without disrupting program goals. The program coordinator also converses with each mentor near the end of the summer to solicit input for student awards for scientific merit. These conversations highlight our exemplary students but also provide opportunities for mentors to share feedback about challenges with program activities.

## Results

### PROGRAM EVALUATION

**Figure 1:**
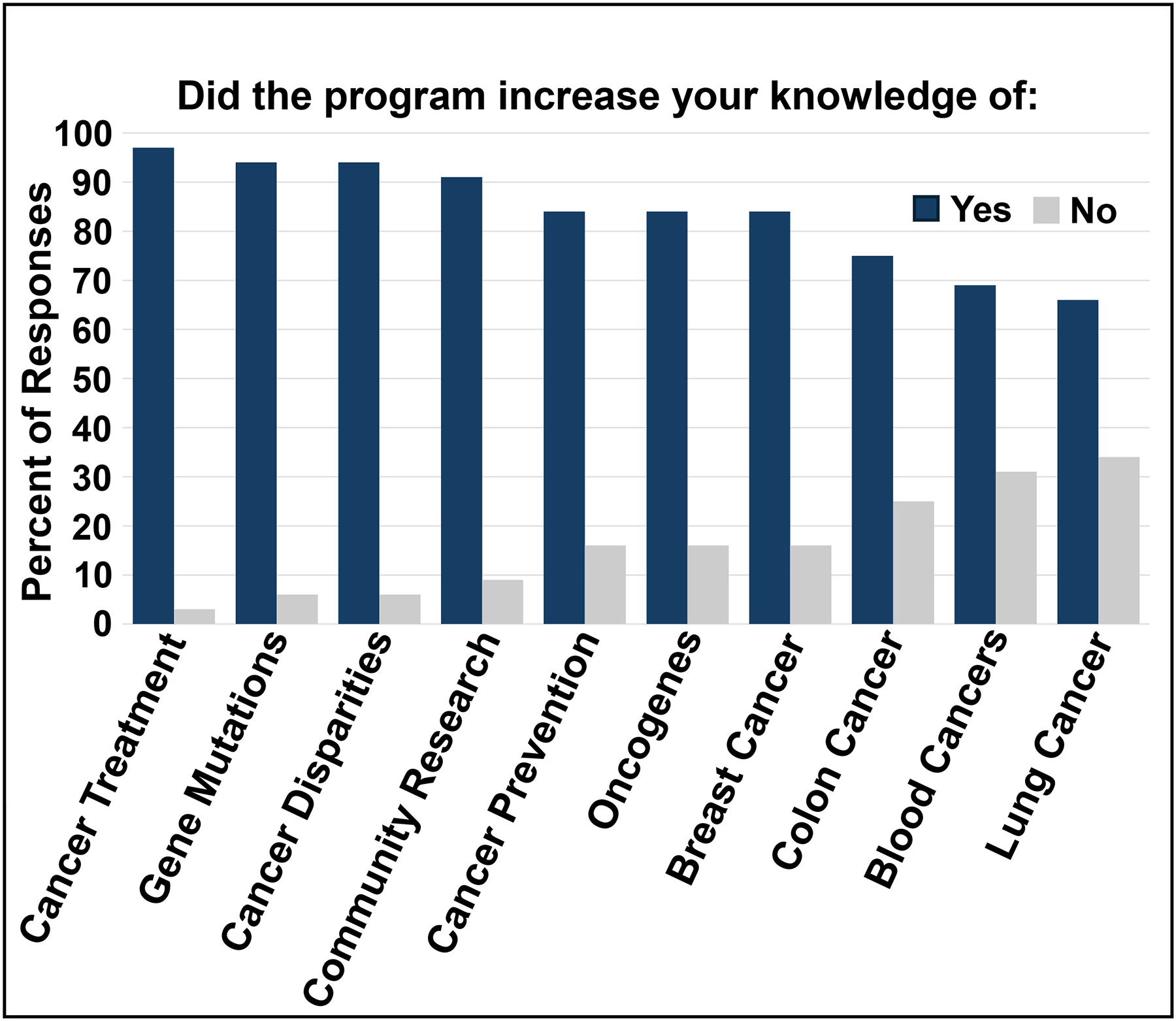
Increases in Student Understanding of Cancer Topics: Percent of students that replied yes (blue bars) or no (grey bars) on the y-axis to increases of understanding the topics on the x-axis.

A post-program survey was conducted by the MERE office distributed via Qualtrics at the end of the summer 2024 Research To Beat Cancer program for both high school students and their mentors. Students were asked to rate their experiences in the program as detailed in methods. About 35% of student participants responded to the post-program survey, a large reduction in survey responses compared to previous years. Survey results noted that 94% of respondents felt the program successfully met their expectations and that they intended to pursue a healthcare career. Overall, 81% of respondents felt the program increased their interest in biomedical or healthcare related careers and enthusiasm for careers in biomedical science. Students highly rated their near peer mentors for their respectful and attentive interactions. Students reported significant knowledge gains in cancer-related topics, with the highest improvements in cancer treatment, gene mutations, cancer disparities, and community engagement (Figure 1).

**Figure 2:**
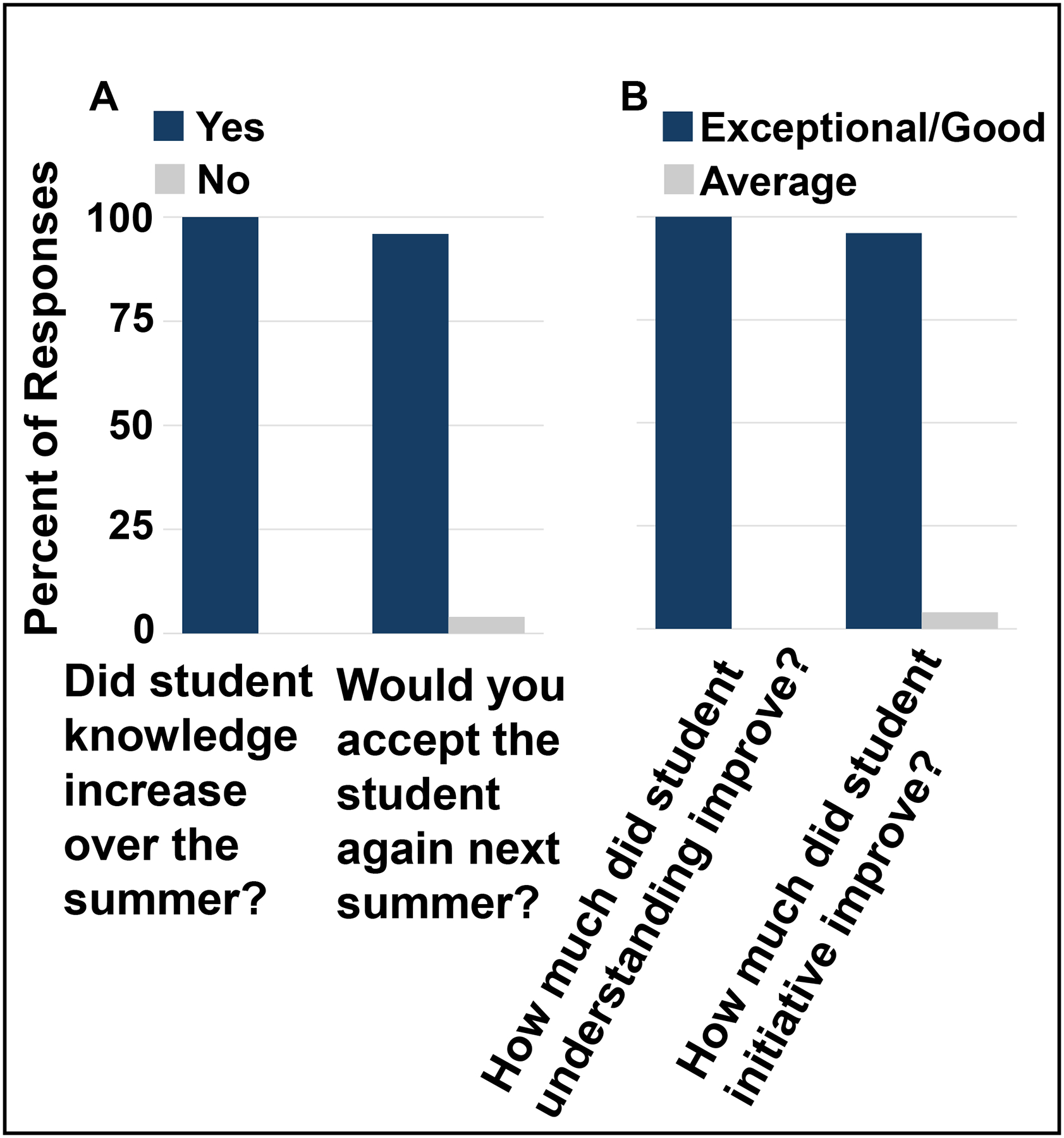
Faculty Mentor Feedback on Student Performance: A) Percent of mentors that replied yes (blue bars) or no (grey bars) on the y-axis to the prompts on the x-axis. B) Percent of mentors rating exceptional or good (blue bars) versus average (grey bars) on the y-axis for student improvements on the x-axis.

Faculty mentors were also surveyed to gauge student success in the program. Approximately 33% of faculty mentors completed the survey, again a large reduction in survey responses compared to previous years. Faculty mentors felt that students were engaged, interacted well with lab personnel, and demonstrated knowledge growth throughout the program. Most felt student knowledge increased over the summer and would accept the student back to their labs the next year (Figure 2A). The majority rated improvement in student understanding and initiative as good to exceptional (Figure 2B).

**Figure 3:**
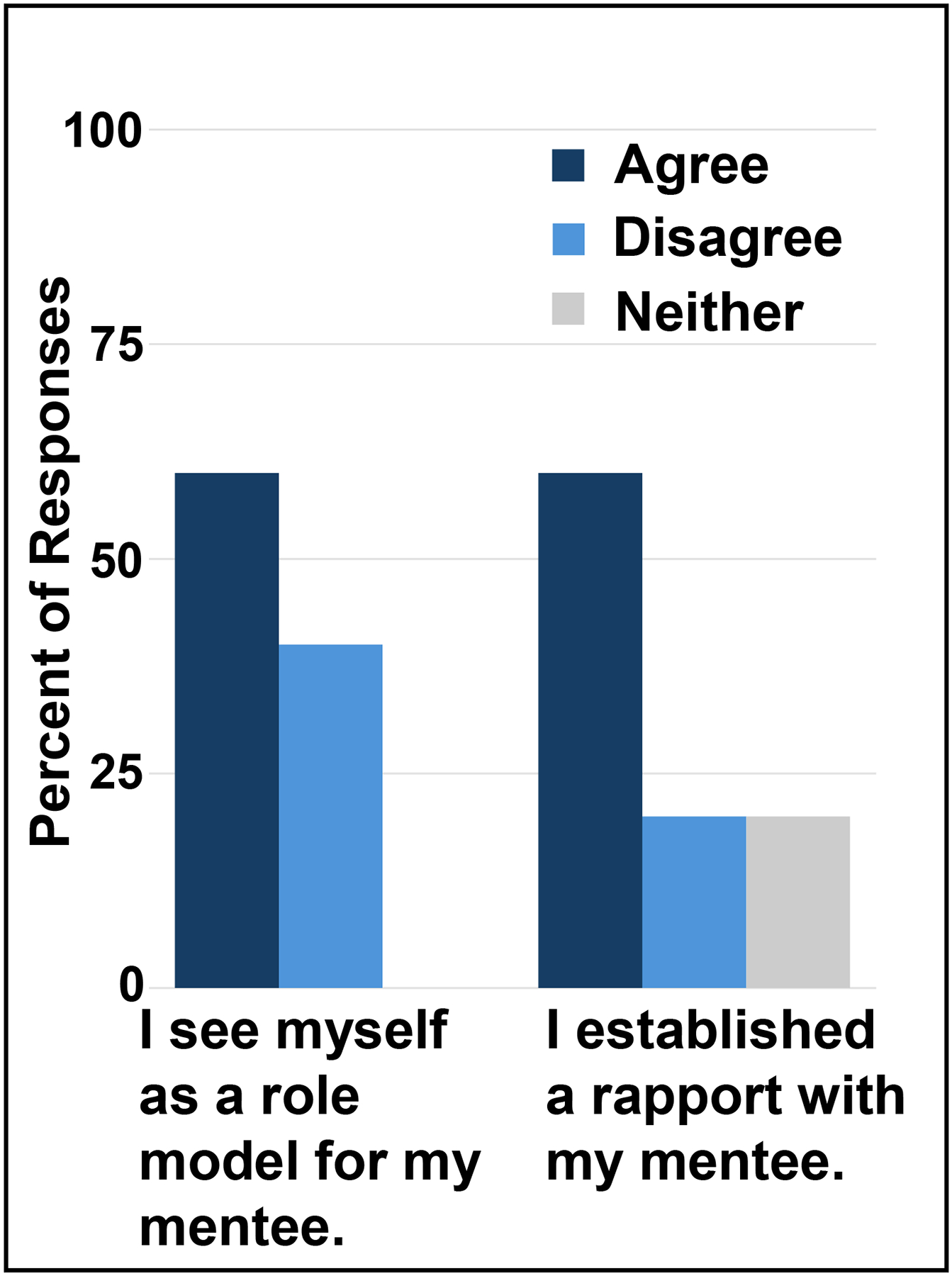
Near Peer Mentor Assessment: Percent of near peer mentors that replied Agree (dark blue bars) Disagree (light blue bars) or neither (grey bars) on the y-axis to prompts on the x-axis.

Roughly 20% of near peer mentors completed their post-program survey. Sixty percent of respondents agreed that they see themselves as role models for their mentees and agreed that they established rapport with their mentees. (Figure 3). This leaves 40% who either disagree or did not agree or disagree, offering an opportunity to enhance the near peer mentoring experience. While many mentors found the experience rewarding and valuable for their careers, in the open-ended question participants recommended clearer role expectations, structured introductory sessions, and incentives to improve engagement.

**Figure 4:**
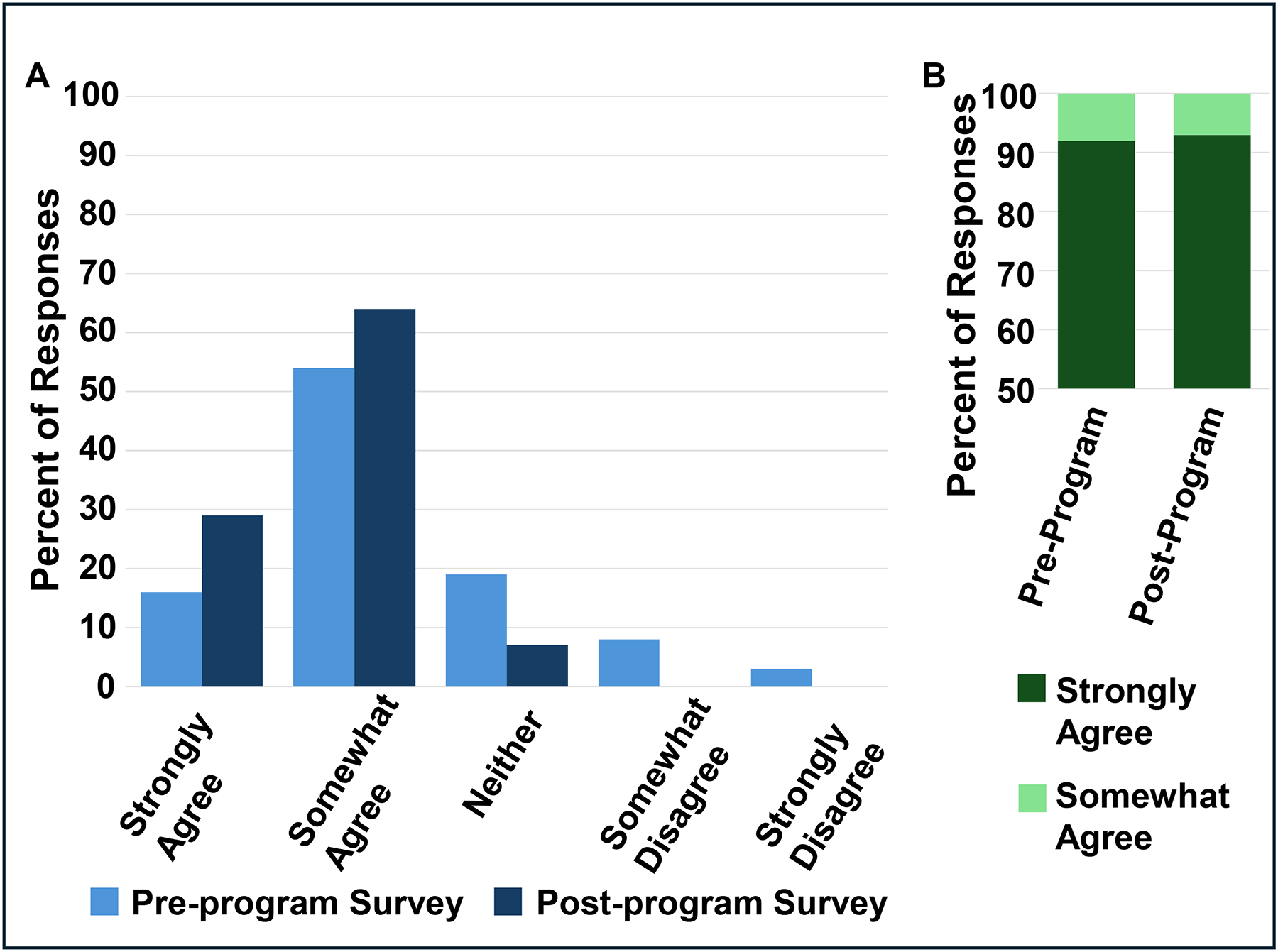

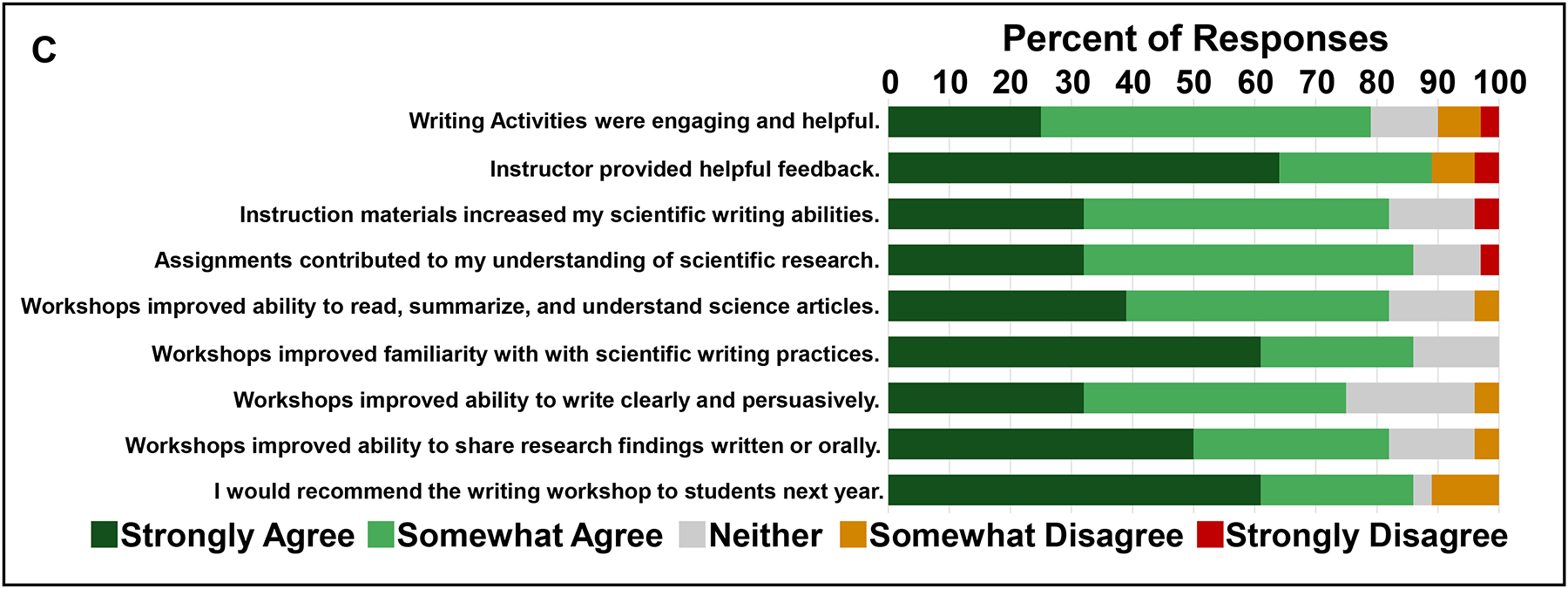
Writing Workshop Assessment: A) Percent of student responses in the pre- (light blue bars) and post-program (dark blue bars) surveys on the y-axis with levels of agreement on the x-axis. B) Percent of student responses of strongly agree (dark green bars) or somewhat agree (light green bars) on the y-axis for pre- and post-program surveys on the x-axis. C.) Survey prompts on the y-axis. Percentage of students responding strongly agree (dark green), somewhat agree (light green), neither agree or disagree (grey), somewhat disagree (orange), and strongly disagree (red) on the x-axis.

A total of 28 out of the 38 students enrolled in the writing workshops completed a pre- and post-program survey conducted by the writing center. In response to the prompt “I am confident in my writing ability”, students showed an increase in strongly and somewhat agree from pre- to post-program surveys (Figure 4A). Students also strongly agreed that learning how to write about science and research were relevant to their studies with a modest increase in strongly agree in the post-program survey (Figure 4B). Most students either somewhat or strongly agreed in the post-program survey that the writing instructors and workshops contributed to student learning (Figure 4C). Some participants provided additional positive feedback, noting that the structured approach—incorporating workshops, small groups, and step-by-step guidance—was especially helpful for those who initially lacked confidence in their writing skills. Peer reviews, guided presentations, and writing exercises were valued for enhancing confidence and offering clear direction. These activities also supported participants in developing their writing styles and improving their understanding of scientific writing.

A voluntary focus group was led by MERE during the final week of the program. There were nine attendees. The focus group findings highlight several strengths of the program. The hands-on experience, interactive lab teams, and near peer mentoring were highly valued by students. The program fostered a comfortable and supportive environment, with students appreciating opportunities such as “Lunch and Learn” sessions and interactive discussions over traditional lectures. Overall, students expressed gratitude for the experience and found it beneficial to their academic and career interests. However, the findings also point to areas for improvement. Students noted challenges with organization and communication, suggesting a need for a centralized platform for program information. Expectations for faculty mentors and lab personnel were sometimes unclear, leading to inconsistencies in mentorship and engagement. Students desired more peer interaction, clearer guidelines for final presentations, and better feedback on manuscripts. Additionally, scheduling conflicts between lab commitments and program activities limited participation in some aspects of the program. Despite these challenges, the program positively influenced students’ career trajectories, increasing their interest in medicine or science and helping them refine their career goals.

The YES program coordinator is actively engaged with students and near peer and faculty mentors prior to and throughout the program. Responsibilities include advertising the upcoming program, soliciting student applications, identifying near peer and faculty members, matching students to mentors and their laboratories, implementing the summer curriculum, and participating in curricular activities. Therefore, the program coordinator built relationships with students and mentors throughout the entire program. This relationship enhanced the communication between the program and stakeholders, resulting in comfort to provide unsolicited feedback. The combination of solicited and unsolicited feedback will result in the following changes to program activities for the upcoming summer.

### RESEARCH IMMERSION

Historically, YES provided a one-day welcome, orientation, and lab safety training to students. Students then arrived at their paired laboratories on day two and participated in scientific research throughout the remainder of the 8-week program. Finally, students were required to submit a formal manuscript and make a final oral presentation of their research projects to the entire group at the end of the summer (Table 1).

Feedback provided by faculty mentors to the program coordinator revealed that students enter the laboratories with varying levels of prior science education, particularly struggling to understand cancer biology. Students were also struggling with the rationale for experimentation and learning the laboratory techniques required to complete their projects. Therefore, many students were not able to successfully complete an experiment over the summer. The requirement of a final manuscript and presentation added additional pressure to students and mentors. Mentors felt that successful completion of a series of experiments were required to create the formal types of manuscripts and presentations they are accustomed to in their careers. Students struggling to complete successful experiments felt they couldn’t meet program and mentor expectations. Feedback also suggested that this stress was exacerbated for students who didn’t feel comfortable asking faculty mentors what might be perceived as naive questions, or didn’t receive the hands-on mentoring they are accustomed to in high school.

Feedback also suggested that an 8-week research immersion was not optimal. Inviting students from a number of different schools in the greater Cleveland area generated conflicts in scheduling due to differences in time-off from school over the summer. Students also participate in academic and athletic training over the summer that is important to their education and development. Faculty mentors also take time off in the summer for academic and personal pursuits.

In response to feedback, we have redeveloped our curriculum for the upcoming summer (Table 2). The program will be reduced to 7-weeks to allow students from all Cleveland area schools to participate during their summer breaks. The shortened timeframe also allows students and faculty mentors who need time for other pursuits to schedule them within the summer but outside of the program dates. In addition, the student orientation will expand from one day to the entire first week of the program. YES will provide a week long cancer biology and research bootcamp to ensure all students start with a solid understanding of cancer biology and the current state of cancer research. The bootcamp will consist of instruction in cancer biology, hands-on laboratory technique training, laboratory safety training, and social/networking activities. The bootcamp should enhance biological understanding, familiarity with laboratory techniques, as well as build student camaraderie before they enter faculty mentors’ laboratories. The bootcamp will end with a luncheon for all students and mentors. This informal setting will celebrate the student’s achievements through week one and provide a thorough introduction of students to their mentors prior to entering the laboratory the following week. These changes should enhance student confidence and comfort with their mentors. Students will participate in cancer research projects with their mentors and additional education and career development activities for the remaining 6-weeks.

To further reduce student and faculty mentor stress, we have made significant changes to the mentoring structure and the expectations of final student products for the next summer. To increase the daily hands-on training of students immersed in laboratories, we will incorporate a faculty and day-to-day mentor dyad for each student. Students will be placed in laboratories of faculty mentors who will oversee the student’s instruction, development, and research. Faculty mentors will identify a day-to-day mentor from their laboratories, senior graduate students or postdocs, who agree to work with the high school students on a daily basis to provide hands-on laboratory technique instruction and ensure progress of the student’s project. This also provides our graduate students and postdocs with an opportunity to mentor others, which is important for their own professional development. In addition, we will provide Mentor Training to all faculty and day-to-day mentor dyads. Mentors can feel overwhelmed by the responsibility of teaching a young student and often fail to recognize that their mentees may have limited knowledge of cancer biology and cancer research techniques. Therefore, we will provide a 2-hour workshop for all mentors to run concurrently with the week 1 cancer biology and research bootcamp (Table 2). The workshop will address three areas: (1) Mentors will learn tips from education experts about how to engage new learners, how to identify different types of learners, and how to create a communicative environment where learners feel comfortable asking questions and clarifying any confusion. (2) Lab specific mentoring will be discussed, including how to use scientific literature relevant to the laboratory to familiarize students with common techniques and experimental designs. By understanding how to identify the knowledge gap between mentor and scholar, the gap can be narrowed to greatly enrich the mentor-learner relationship. (3) Mentors will be provided with an overview of the YES curriculum, and specific program expectations for the student’s experience and final presentations.

YES, will no longer require a final scientific manuscript of student projects. Students will be required to submit a scientific abstract and final oral presentation succinctly describing what the student accomplished in the laboratory, focusing on what they learned from and enjoyed most about the program. Mentors and students will be provided with a template for the abstract and final presentation that will focus on the scientific rationale of the experiment, the methods used, any results obtained, and what the student learned. This should eliminate the stress of successfully completing a line of experimentation so that the student and mentor feel they have results to share. It will provide an opportunity for the students to communicate why they did their project, how they were conducting their experiments, any results they obtained, and what they learned over the summer.

### WRITING WORKSHOPS

Historically, YES provided the writing workshop only to select students that require additional training in reading comprehension, writing, and presentation skills. Feedback from students indicated that all students felt they would benefit from additional writing instruction. Some students who did not participate in the writing workshop also did not receive feedback from their mentors about their abstracts, manuscripts, or presentations. Also, students indicated they are preparing to apply for college or job opportunities and felt they would benefit from instruction in written and oral communication for writing college essays, job applications, and preparing for interviews. Feedback also indicated that students, mentors, and writing instructors disliked the final manuscript requirement as all struggled to meet expectations when experiments failed, or the results were not clear or unexpected.

In response to feedback, we have made significant changes to the structure and function of the writing workshop. This summer all students will be engaged in the writing workshop, instead of a select group that is identified as needing additional instruction. As discussed above, the final manuscript will no longer be required. Instead, students will generate a portfolio of work that meets program requirements but also benefits them in their educational and professional pursuits. The new portfolio will consist of a resume, a college essay or job application cover letter, a scientific abstract of their project, and a brief description of their summer experience. The purpose will be to train the students to present their work succinctly in a manner that is accessible to lay audiences. The brief description of their summer experience will mimic an “elevator pitch” and can used as a written transcript for their oral presentation. The goal is to teach the students how to effectively communicate, introduce them to scientific writing, and provide them with valuable resources they can use for college or job applications.

### CAREER DEVELOPMENT ACTIVITIES

Historically, the YES program provided lunch and learn, career café, near peer mentoring, and writing workshops three times a week with some combined activities lasting nearly 3 hours at a time (Table 1). Feedback from students indicated that it was difficult to maintain focus for long stretches of time and large blocks of time away from the laboratory was impeding experimental progress. Feedback provided from mentors indicated that some of the students did not have a lunch nor the funds to purchase one. Students from some of our high schools are accustomed to free lunches and we learned that mentors were purchasing lunch for those who couldn’t afford one on days when there was no lunch and learn session.

In response to this feedback, we have adjusted the timing and frequency of our career development activities. YES will provide career development activities each day from noon-1pm with lunch provided to all (Table 2). This will eliminate the large blocks of time in these activities that challenge student focus. It will also allow students to consistently be present for more time in the laboratories when research is active. Finally, the program coordinator will be present at each activity interacting with students to provide a daily opportunity for relationship building and receiving feedback.

### SURVEYS

Surveys for the 2024 YES program were limited due to a low response rate. Next summer we will provide built-in time within the first week of the program and during near peer mentoring sessions at the end of the program to allow for students and near peer mentors to complete surveys. We will also streamline communications between the evaluation team and the program coordinator to ensure all participants are aware of and engaged in feedback opportunities.

## Discussion

Cancer is a major public health concern and area of research as it is the second most common cause of death in the United States totaling about 1,680 deaths per day in 2024.^23^ Growing the biomedical research and healthcare workforce is critical to combating the rise in cancer incidence associated with the overall aging of the United States population. Therefore, providing students with opportunities and access for future careers in STEM has been a national priority with numerous programs developed to improve mastery at all levels of education across the United States.^24–26^ Summer STEM internships increase college readiness in STEM disciplines through hands-on activities that excite students about research careers.^27^ Internships impact career trajectories, preparing students for the rigors of college STEM courses by increasing participant confidence in reading and understanding primary scientific articles.^28,29^ Interpersonal factors, such as interaction with educators and peers, impacts early career decisions of students.^30^ Indeed, interns often feel closer to, and better mentored by near peer mentors than by faculty.^31^ The Case CCC’s YES program, funded by the National Cancer Institute, was created to promote broad interest in pursuing a career in biomedical research via early intervention strategies that inspire student interest, help envision research as a career path, and strengthen practical research and career skills. Importantly, increasing the cancer awareness of youth may also enhance cancer prevention and screening activities that contribute to lower cancer rates.^32^

Evaluation suggests the YES program is meeting its goals of increasing student understanding of cancer biology and research while enticing them to consider future careers in biomedical research and healthcare. The YES program is generally providing a positive experience to students and mentors. While the post-program survey response rate was low for the summer of 2024 compared to previous years, the results from prior years have been consistently positive and align closely with this year’s feedback, suggesting that the overall student and mentor experience has remained strong over time.^1,12–14^

The evaluation findings reinforce the importance of integrating mixed methods approaches to fully capture the complexities of student experiences, as highlighted in prior research on pathway programs. While formal survey data provide valuable longitudinal insights, lower response rates can create concerns about the extent to which responses reflect the full range of student perspectives. Mixed methods research underscores the value of triangulating data from multiple sources—including structured surveys, mentor observations, and student reflections—to create a more comprehensive assessment framework.^17,18,20^ Incorporating more diverse feedback channels, such as structured mentor debriefs and real-time student reflections, help mitigate response rate fluctuations while ensuring that program improvements are informed by a broader and more representative dataset. This approach is particularly relevant for pathway programs aimed at increasing STEM participation, as it allows evaluators to capture not only immediate student experiences but also longer-term shifts in confidence, career interests, and self-efficacy in biomedical research.^7,10,15^

We acknowledge that the response rate for 2024 makes it challenging to determine if survey respondents were primarily those with the most positive experiences, while those with more mixed experiences opted to provide informal feedback instead. Integrating both formal and informal feedback mechanisms in program evaluation is essential for capturing the full range of student experiences. Formal surveys provide structured, comparable data over time, helping track trends and measure the effectiveness of curricular changes. However, informal feedback through focus groups, direct conversations, or other channels captures perspectives that might not emerge in surveys, particularly from students who may feel less inclined to participate in formal evaluations. As mentioned above, this approach is well supported by mixed methods research, which demonstrates that leveraging quantitative and qualitative data produces a more comprehensive and actionable evaluation.^17^ Together, these approaches provide a thorough understanding of student experiences and ensure that curricular changes are made that reflect the full range of student needs. Furthermore, establishing a collaborative partnership between an external evaluation group, such as the MERE office, and internal program stakeholders enhances responsiveness by ensuring that both systematically analyzed data and real-time feedback inform curricular revisions. This strategy aligns with the principles of utilization-focused evaluation, which emphasizes the active involvement of stakeholders in both data collection and decision-making processes to drive meaningful improvements.^18,20^ This collaborative approach not only strengthens the validity of the data collected but also facilitates timely curricular adjustments.

Our results also demonstrate the critical importance of routine informal discussions between stakeholders and a program coordinator. In the case of YES, our program coordinator is accessible to all stakeholders and knows the student participants well from the application process and through regular interaction at program activities. Having been a part of the program for multiple years, the program coordinator is also familiar to our participating mentors. An approachable program coordinator can act as an advocate for both students and mentors, collecting feedback in real-time to enact program changes that benefit all. It is also important to empower the program coordinator to make immediate changes or execute changes for the future to enhance the participants experience in the program.^33^

## Conclusion

The YES program utilizes a mix of formal and informal mechanisms to solicit feedback from stakeholders for program evaluation and enhancement. YES relies on an external body of evaluation and analysis for formal surveys, while empowering its program coordinator to suggest or make changes in response to informal feedback. For the upcoming summer, the YES program curriculum has been modified to enhance not only student and mentor experiences but also the process for soliciting formal and informal feedback. Evaluation of the changes made will be important for the evolution of the program into the future.

## Figures and Tables

**Table 1:** Past YES Program Activities

Week 1	Monday	Tuesday	Wednesday	Thursday	Friday
	Student Orientation Lab Safety Training Mentor Pairing	Same as below	Same as below	Same as below	Same as below
Weeks 2–7	Monday	Tuesday	Wednesday	Thursday	Friday
9–10am	Laboratory Immersion				
10–11am					
11–12pm					
12–1:30pm	Writing Workshop (select students)	Lunch and Learn (Lunch provided)	Near-Peer Mentoring (Lunch provided)	Lunch and Learn (Lunch provided)	Writing Workshop (select students)
1:30–3:00pm	Laboratory Immersion			Career Café	Science in the News
3–4pm				Laboratory Immersion	
4–5pm					
4–5pm					
Week 8	Monday	Tuesday	Wednesday	Thursday	Friday
	Same as above	Same as above	Same as above	Same as above	End of summer symposium with student presentations

**Table 2:** Future YES Program Activities

Week1 Bootcamp	Monday	Tuesday	Wednesday	Thursday	Friday
9–12pm	Orientation and Welcome	Discussion of Cellular Biology	Discussion of Cancer Biology	Discussion of Cancer Treatment	Discussion of Cancer Prevention
12–2pm	Cohort Building Lunch & Networking Activities Students only			Mentor Training (faculty & near peer mentors)	Student-Mentor Luncheon
2–5pm	Lab Safety Training	Lab Technique Training	Lab Technique Training	Lab Technique Training	Students Go to Laboratories
Weeks 2–7	Monday	Tuesday	Wednesday	Thursday	Friday
9–12pm	Laboratory Immersion				
12–1pm (Lunch provided)	Writing Workshop & Lunch	Lunch and Learn	Writing Workshop & Lunch	Career Café & Lunch	Near-peer Mentoring & Lunch
1–5pm	Laboratory Immersion				
